# Natural Selection Shapes Maintenance of Orthologous sRNAs in Divergent Host-Restricted Bacterial Genomes

**DOI:** 10.1093/molbev/msab202

**Published:** 2021-07-02

**Authors:** Margaret W Thairu, Venkata Rama Sravani Meduri, Patrick H Degnan, Allison K Hansen

**Affiliations:** 1 Department of Entomology, University of California, Riverside, Riverside, California, USA; 2 Department of Bacteriology, University of Wisconsin, Madison, Madison, Wisconsin, USA; 3 Department of Microbiology and Plant Pathology, University of California, Riverside, Riverside, California, USA

**Keywords:** small RNA, antisense small RNA, small genomes, *Carsonella*, *Bactericera cockerelli*, *Diaphorina citri*

## Abstract

Historically it has been difficult to study the evolution of bacterial small RNAs (sRNAs) across distantly related species. For example, identifying homologs of sRNAs is often difficult in genomes that have undergone multiple structural rearrangements. Also, some types of regulatory sRNAs evolve at rapid rates. The high degree of genomic synteny among divergent host-restricted bacterial lineages, including intracellular symbionts, is conducive to sRNA maintenance and homolog identification. In turn, symbiont genomes can provide us with novel insights into sRNA evolution. Here, we examine the sRNA expression profile of the obligate symbiont of psyllids, *Carsonella ruddii*, which has one of the smallest cellular genomes described. Using RNA-seq, we identified 36 and 32 antisense sRNAs (asRNAs) expressed by *Carsonella* from the psyllids *Bactericera cockerelli* (*Carsonella-BC*) and *Diaphorina citri* (*Carsonella-DC*), respectively*.* The majority of these asRNAs were associated with genes that are involved in essential amino acid biosynthetic pathways*.* Eleven of the asRNAs were conserved in both *Carsonella* lineages and the majority were maintained by selection. Notably, five of the corresponding coding sequences are also the targets of conserved asRNAs in a distantly related insect symbiont, *Buchnera.* We detected differential expression of two asRNAs for genes involved in arginine and leucine biosynthesis occurring between two distinct *Carsonella-BC* life stages. Using asRNAs identified in *Carsonella*, *Buchnera*, and *Profftella* which are all endosymbionts, and *Escherichia coli*, we determined that regions upstream of these asRNAs encode unique conserved patterns of AT/GC richness, GC skew, and sequence motifs which may be involved in asRNA regulation.

## Introduction

Small RNAs (sRNAs) have emerged as key players in bacterial gene regulation of virtually all aspects of cellular physiology (Nitzan et al. 2017). Further, sRNA regulators are metabolically cheap and allow bacteria to rapidly respond to changes in the environment (Beisel and Storz 2010). The origins of sRNAs in bacteria are facilitated by processes similar to those observed for protein coding genes, such as de novo emergence, gene duplication, and horizontal gene transfer (Dutcher and Raghavan 2018). Nevertheless, there is still an incomplete understanding of sRNA evolution and function relative to protein coding genes, and many of the evolutionary studies to date are biased toward sRNAs found in model, free-living bacterial species, which include *Escherichia coli*, *Salmonella enterica*, *Citrobacter freundii*, *Klebsiella pneumoniae*, *Serratia marcescens*, and *Yersinia enterocolitica* (e.g., Skippington and Ragan 2012; Cerutti et al. 2017; Chen et al. 2017; Kacharia et al. 2017). Moreover, it is often difficult to identify sRNA homologs among divergent bacterial taxa due to 1) the fast rate at which sRNA sequences and structures evolve, 2) high false-positive prediction rates, and 3) genome rearrangements (Dutcher and Raghavan 2018). Examination of bacterial lineages that possess genomic characteristics that are conducive to sRNA maintenance and homolog identification across distantly related species (Ruwe and Schmitz-Linneweber 2012; Ro et al. 2013; Hansen and Degnan 2014; Thairu et al. 2018; Thairu and Hansen 2019a) can provide us with novel insights into bacterial sRNA evolution (Thairu and Hansen 2019b).

The genomic architecture of many bacterial symbiont taxa that are host-restricted and other small genomes, such as eukaryotic organelles, helps facilitate sRNA maintenance and identification at deep evolutionary distances (Thairu and Hansen 2019b). For example, many host-restricted genomes are not able to recombine or participate in horizontal gene transmission, which generally results in highly syntenic genomes even after millions of years of divergence (McCutcheon and Moran 2011; Moran and Bennett 2014). This may result in a greater degree of sRNA conservation in these host-restricted genomes compared with free-living bacteria where recombination and horizontal gene transfer frequently disrupt sRNAs in related free-living bacterial taxa (Dutcher and Raghavan 2018). A second characteristic of host-restricted symbiont and mitochondrial genomes that may facilitate the identification of sRNA homologs compared with free-living bacteria is high coding density (Thairu and Hansen 2019b). High coding density may influence the types of sRNAs present in these host-restricted bacterial genomes. Specifically, most bacterial symbiont and organelle sRNAs that have been detected to date are *cis*-acting and transcribed antisense to their target coding sequences (Thairu and Hansen 2019b). *Trans-*acting sRNAs are fewer in number among symbiont genomes likely as a result of genome-wide reductions of noncoding regions and the loss of RNA chaperone proteins that are frequently required for *trans*-acting sRNAs in free-living bacteria (Thairu and Hansen 2019b). *Trans-*acting sRNAs are encoded in genomic locations that are far removed from their target, and often share only partial complementarity to their target (the seed region), whereas *cis*-acting, antisense sRNAs (asRNAs) have perfect complementarity to their target and often do not require an RNA chaperon protein (Thomason and Storz 2010; Georg and Hess 2011; Storz et al. 2011; Millar and Raghavan 2021). As a consequence, *trans*-acting sRNAs may evolve more rapidly than *cis*-acting sRNAs, especially in their seed region, because of their degenerate complementarity and ability to evolve additional new targets over time (Dutcher and Raghavan 2018).

Another characteristic of host-restricted bacterial genomes that may influence the evolution of proto-sRNAs is a pervasive adenine and thymine (AT) bias (Thairu and Hansen 2019b). Many obligate intracellular bacterial symbionts experience severe population bottlenecks and deletion of DNA replication and repair enzymes that often result in dramatic genomic changes, such as a reduction in genome size and AT bias (McCutcheon and Moran 2011; Moran and Bennett 2014). It has previously been proposed by Lloréns-Rico et al. (2016) that the number of asRNAs in genomes is positively related to genomic AT content and that these antisense transcripts are primarily the result of spurious transcription sites based on the “TANAAT” motif and therefore are just transcriptional noise. This latter study however did not investigate if other conserved promoter sites or motifs are present upstream of asRNAs especially in related host-restricted lineages with AT rich genomes. It is possible that sRNA promoter sites are not the same as for coding sequences (e.g., “TANAAT”) and/or that they are not conserved across large evolutionary distances, as assumed in Lloréns-Rico et al. (2016).

A diversity of host-restricted bacterial symbionts are prevalent among the most species-rich group of animals, the insects (Douglas 2011; Flórez et al. 2015; Hammer and Bowers 2015; Moran et al. 2019; Frago et al. 2020). Many of these symbionts’ genomes have been sequenced and possess genomic characteristics that facilitate sRNA conservation and are AT biased (McCutcheon et al. 2019). For example, the nutritional aphid symbiont *Buchnera aphidicola* (Gammaproteobacteria) (hereafter *Buchnera*), is the best characterized symbiont of sap-feeders within the insect Order Hemiptera. There is increasing evidence that this obligate aphid symbiont regulates its own gene expression using regulatory sRNAs that are conserved across *Buchnera* lineages that diverged ≥65 Ma (Hansen and Degnan 2014; Thairu et al. 2018; Thairu and Hansen 2019a, Blow et al. 2020). Within the hemipteran superfamily, Psylloidea, all psyllid members have evolved an obligate symbiotic relationship with the gammaproteobacterium, *Carsonella ruddii* (hereafter *Carsonella*) (Thao et al. 2000). *Carsonella*, like many hemipteran symbionts, including *Buchnera*, convergently evolved to be nutritional symbionts of their sap-feeding hosts; supplementing the psyllid’s diet with amino acids which are deficient in their plant sap diet. *Carsonella* has one of the smallest insect symbiont genomes sequenced to date of ∼166 kb (Moran and Bennett 2014; NCBI Genomes 2020), and displays extremely high gene density with many genes overlapping one another (Nakabachi et al. 2006). In this study, we analyze two lineages of *Carsonella* from two different psyllid families*.* The first from the potato psyllid, *Bactericera cockerelli* from the family Triozidae (hereafter: *Carsonella-BC*), a pest of Solanaceous crops and the vector of “*Candidatus* Liberibacter psyllaurous,” which is associated with psyllid yellows disease (Hansen et al. 2008). The second from the Asian citrus psyllid, *Diaphorina citri*, from the family Liviidae (hereafter: *Carsonella-DC*), the vector of “*Candidatus* Liberibacter asiaticus,” which is associated with citrus greening disease (Jagoueix et al. 1994). *Diaphorina citri* also harbors the co-obligate bacterial intracellular endosymbiont *Profftella armature* (Betaproteobacteria) (hereafter *Profftella*) in the syncytial region of the bacteriome (the specialized organ that houses endosymbiotic bacteria which is mainly found within insects) (Subandiyah et al. 2000; Nakabachi et al. 2013). Although *Profftella’s* genome is larger than *Carsonella’s*, *Profftella* still has a reduced genome (∼464 kb), and serves as a defensive symbiont for the psyllid by producing the polyketide toxin diaphorin, which is predicted to protect against various natural enemies of its psyllid host (Nakabachi et al. 2013; Szebenyi et al. 2018; Yamada et al. 2019). In addition, *Profftella*, also contains genes related to hemolysin, riboflavin, biotin, and carotenoid biosynthesis (Nakabachi, Piel, et al. 2020).

To build a deeper understanding of sRNA evolution, we address the following questions: 1) Are conserved sequence motifs or nucleotide composition patterns present within regions upstream of expressed sRNAs in genomes of related and unrelated host-restricted symbionts when compared with a free-living relative such as *E. coli*? The presence of conserved promoter sites may provide insight into potential sRNA regulatory mechanisms in these stable, host-restricted genomes. Focusing on *Carsonella*, we also address the question: 2) What is the likelihood that conserved sRNA homologs identified in the psyllid symbiont, *Carsonella* are functional? Evidence of natural selection maintaining conserved sRNA homologs and differential expression provides further support that a sRNA is functional and not just transcriptional noise.

## Results

### Widespread Expression of Antisense sRNAs in *Carsonella* Lineages

sRNAs in host-restricted genomes, including *Buchnera* and organelles, have previously been found to be differentially expressed between host developmental stages (Itaya et al. 2008; Hansen and Degnan 2014; Ma et al. 2016; Thairu et al. 2018; Thairu and Hansen 2019a). To capture a wide range of endosymbiont sRNAs, we sampled across various life stages for both *Bactericera cockerelli* and *Diaphorina citri*. Psyllids are hemimetabolous insects that undergo five developmental instars before adulthood. For *B. cockerelli*, two life-stage-specific samples were collected: 1) Dissected adult insect cells that harbor *Carsonella* called bacteriocytes (males and females; gravid and nongravid) (BC-A1, BC-A2, BC-A3), 2) whole body fifth instar nymphs that harbor bacteriocytes (BC-N1, BC-N2, BC-N3). For these life-stage-specific samples, three biological replicates of ∼60 psyllids (approx. 30 males and 30 females per sample) were collected. A third sample that contains a mixture of whole-body 1st–5th instar *B. cockerelli* nymphs and *B. cockerelli* adults (males and females; gravid and nongravid) (BC-All) was also collected. For *D. citri*, only one type of sample was collected; a mixture of whole-body 1st–5th instar nymphs and adults (males and females; gravid and non-gravid) (DC-All). *Diaphorina citri*, is an invasive species and is reared under strict quarantine protocols and therefore we had limited access to samples. The mixture of life stages for specific samples from both psyllid species (BC-All and DC-All) consisted of a population of ∼20 psyllids per life stage.

**Fig. 1.**
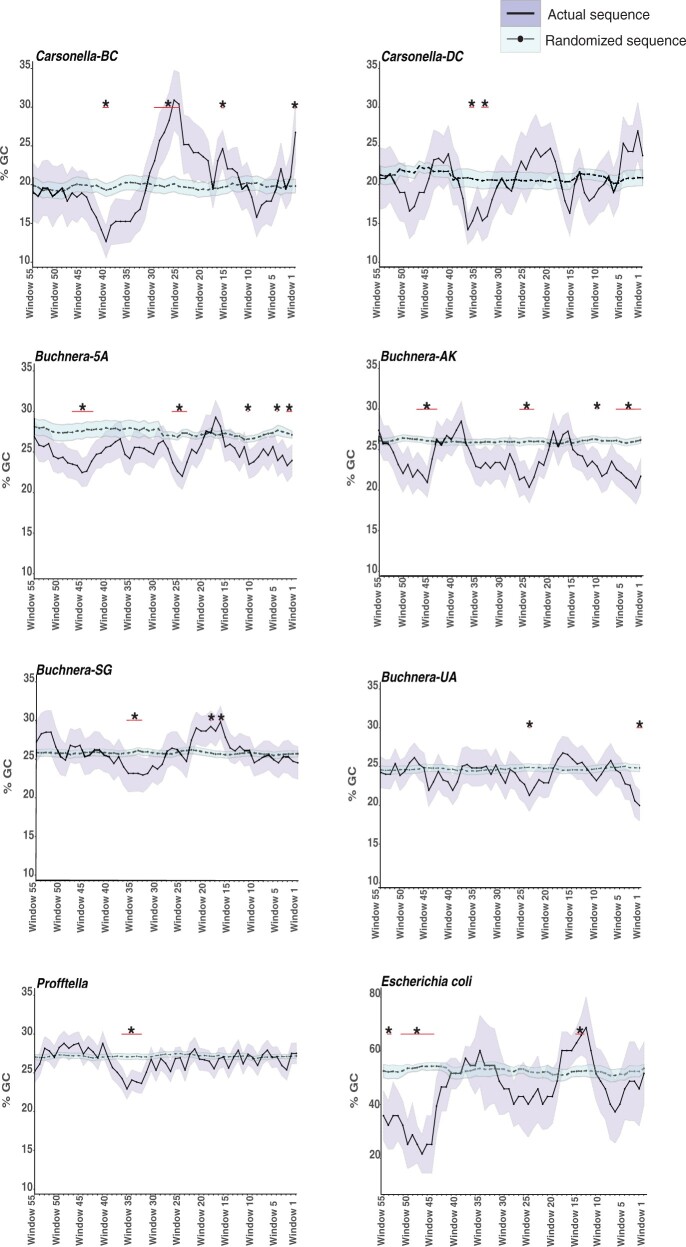
Sliding window analysis of the nucleotide content found in the 61 nt upstream region of expressed asRNAs in symbiont lineages and *Escherichia coli*. The shaded region around each line represents the standard error. Windows that have a significant nucleotide enrichment (*P* < 0.05) compared with a randomized sequence are marked by a *. The red line under the * indicates how many windows are significant within that region. See supplementary table 6, Supplementary Material online for sample size for each.

For this study, the presence of putative sRNAs that are expressed antisense to the gene (asRNAs), sRNAs expressed within the untranslated regions of genes (UTR sRNAs), and sRNAs identified within the intergenic spacer regions (intergenic sRNAs) were investigated in *Carsonella* and *Profftella* (see Materials and Methods for further details). Using strand-specific RNA-seq of the sRNA enriched, size-selected RNA fraction (≤250 nt), the sRNA expression profiles of the two *Carsonella* lineages were determined. On average each of the seven libraries yielded ∼33 million trimmed, high quality reads (table 1). However, the proportion of each library that mapped to the bacterial endosymbiont genomes varied from 8% to ≤1%. As expected, the dissected adult bacteriocytes (BC-A1-3) had the highest proportion of *Carsonella* to insect reads. The samples derived from whole bodies of nymphs or adults (BC-N, BC-All, and DC-All) had a greater fraction of insect reads to symbiont reads. In *D. citri*, the proportion of reads mapping to *Profftella* outnumbered *Carsonella-DC* ∼20:1, which may be due to the higher *Profftella* titer present in all psyllid life stages (Dossi et al. 2014) (table 1). Regardless, sufficient read coverage was obtained to identify the majority of expressed putative sRNAs from both the *Carsonella* and *Profftella* genomes (table 1 andsupplementary fig. 1, Supplementary Material online).

**Table 1.** Summary of RNA-Seq Data from *Bactericera cockerelli* and *Diaphorina citri* Samples.

Samples^a^	Total Number of Reads	Reads after Quality Screen and Adapter Trimming	Reads Aligning to Genome	Average Genome Coverage
*Carsonella-BC* (173,802 bp)				
BC-A1	3.81 × 10^7^	3.30 × 10^7^	2.24 × 10^6^	974
BC-A2	4.32 × 10^7^	3.59 × 10^7^	1.80 × 10^6^	782
BC-A3	4.42 × 10^7^	3.63 × 10^7^	1.91 × 10^6^	829
BC-N1	4.49 × 10^7^	2.96 × 10^7^	4.00 × 10^4^	17
BC-N2	5.92 × 10^7^	2.97 × 10^7^	2.61 × 10^4^	11
BC-N3	5.49 × 10^7^	3.78 × 10^7^	2.56 × 10^4^	11
BC-All	5.75 × 10^7^	3.54 × 10^7^	7.41 × 10^4^	32
*Carsonella-DC* (174,014 bp)				
D-All	4.64 × 10^7^	2.92 × 10^7^	1.67 × 10^5^	73
*Profftella* (464,857 bp)				
			2.25 × 10^6^	368

a BC-A1-3 and BC-N1-3 represent *B. cockerelli* adult and fifth instar nymph life-stage samples, respectively. BC-All and D-All are pooled samples of all *B. cockerelli* and *D. citri* life stages.

Within both lineages of *Carsonella*, only asRNAs were predicted from the expression data. *Carsonella* genomes are characterized by having high gene density with very few intergenic spacer regions (Sloan and Moran 2012). In turn, this high gene density characteristic likely influences the lack of observed expressed intergenic sRNAs. All seven *Carsonella-BC* samples (BC-A1-3, BC-N1-3, and BC-All) were used initially to determine lineage-specific sRNAs. From these samples, 36 asRNAs that are predicted to target 27 CDSs were identified (supplementary table 1, Supplementary Material online). Analysis of the *Carsonella-DC* reads yielded 32 asRNAs that were predicted to target 27 CDSs (supplementary table 2, Supplementary Material online).

PANTHER GO functional gene list analysis (Mi et al. 2019) was used to determine GO pathways associated with the predicted target CDS of identified sRNAs. For the predicted CDS targets of expressed asRNAs found within *Carsonella-BC*, 13 GO pathways were identified. Seven of the pathways were associated with the biosynthesis of the essential amino acids: arginine, chorismate, histidine, isoleucine, leucine, lysine, and valine (table 2). Similar to *Carsonella-BC* samples, the majority (7/10) of the identified GO pathways for *Carsonella-DC* were associated with the biosynthesis of the essential amino acids: arginine, chorismate, isoleucine, leucine, lysine, threonine, and valine (table 2).

**Table 2.** GO PANTHER Pathways of the Predicted CDSs for sRNAs Found in *Carsonella-BC* and *Carsonella-DC.*

	Predicted CDS of Expressed sRNA	
Pathway Associated with Predicted sRNA Target	*Carsonella-BC*	*Carsonella-DC*
5-Hydroxytryptamine degradation	*putA*	—
Alanine biosynthesis	*ilvE*	—
Arginine biosynthesis	—	*argH*
	*carA-carB*	*carA*
	*carB*	*carB*
ATP synthesis	*atpA*	*atpA*
Chorismate biosynthesis	*aroA*	—
	*aroC*	*aroC*
De novo purine biosynthesis	*purA*	—
De novo pyrimidine ribonucleotides biosynthesis	*carA-carB*	*carA*
	*carB*	*carB*
Histidine biosynthesis	*hisD*	—
Isoleucine biosynthesis	*ilvE*	*ilvD*
Leucine biosynthesis	*ilvE*	*leuD*
	*leuC*	*leuC*
Lysine biosynthesis	*ilvE*	*lysC*
	*lysA*	—
	*dapF*	—
Pentose phosphate pathway	*tktA*	*tktA*
Threonine biosynthesis	—	*lysC*
Valine biosynthesis	*ilvE*	*ilvD*

Note. “—” not targeted in *Carsonella* taxa. Underlined pathways are related to essential amino acid biosynthesis.

### *sRNAs* Are Conserved within *Carsonella* Lineages

The presence of conserved sRNAs across divergent lineages can provide evidence in support of the hypothesis that the sRNAs are selectively maintained for regulatory and/or structural functions. In both *Carsonella-BC* and *Carsonella-DC*, asRNAs were predicted to target the CDSs: *aroC*, *atpA*, *atpD*, *carA*, *carB*, *clpX*, *dnaK*, *gidA*, *grepE*, *leuC*, and *prfA* (supplementary tables 1 and 2, Supplementary Material online). To identify if the predicted asRNAs were orthologous to one another, the following criteria from Hansen and Degnan (2014) were used: 1) the sRNA is a discreet transcript at a specific location within the gene, 2) the sRNA transcript was predicted using the Rockhopper optimized thresholds as described in Materials and Methods, and 3) the regions overlap one another. Using these criteria, 11 of the asRNAs, including those within *aroC*, *atpA*, *atpD*, *carA*, *carB*, *clpX*, *gidA*, *grepE*, *leuC*, and *prfA* were conserved between both *Carsonella-BC* and *Carsonella-DC* (table 3). The number of conserved sRNAs identified represents a significant proportion of the total sRNAs that were identified (one-tailed *z*-proportion test, *Carsonella-BC: z* = 2.7, *P *<* *0.01; *Carsonella-DC: z* = 1.9; *P *<* *0.01).

**Table 3.** Sequence and Structural Analysis of Conserved asRNAs.

ML Distance of AA Sequences										
	Alignment Length^a^	sRNA Region^b^	Non-sRNA Region of Protein	ΔG^c^ (BC/DC)^d^	No. of Compensatory Changes (BC/DC)	No. of Aligned *Carsonella* Sequences	ΔG (ALL)^e^	No. of Compensatory Changes (ALL)	No. of Shared Pairs	% of Shared Pairs
*aroC*	89	*0.197*	0.208	−29.4	2	11	−7.6	1	0	0%
*atpA_1*	111	*0.132*	0.229	−42.6	5	11	−22.8	4	18	53%
*atpA_2*	178	*0.117*	0.238	−63.4	5	11	−30.1	5	19	36%
*atpD*	240	*0.074*	0.107	−90.2	6	11	−55.4	7	39	48%
*carA-carB*	329	0.487	0.458	−126.8	9	9	−51.3	10	14	13%
*carB*	107	0.437	0.312	−26.8	2	9	−6.3	5	5	15%
*clpX*	138	*0.170*	0.220	−42.2	6	11	−13.2	2	5	11%
*gidA*	150	0.373	0.231	−45.0	5	11	−13.7	4	0	0%
*grpE*	145	*0.361*	0.569	−44.1	6	11	−10.0	6	5	11%
*leuC*	193	*0.123*	0.265	−65.4	7	11	−30.3	7	0	0%
*prfA*	143	*0.122*	0.435	−37.3	2	11	−18.1	2	16	39%

a Length of overlapping sRNA region with additional 15 nt upstream and downstream in *Carsonella-BC* and *Carsonella-DC*.

b Values in italics are significantly more conserved than surrounding protein coding region in one-tailed *t*-test, *P *<* *0.01.

c Thermodynamic ensemble prediction (kcal/mol) from RNAalifold.

d BC/DC = *Carsonella-BC* and *Carsonella-DC*.

e ALL = all aligned *Carsonella* strains.

To further determine if these orthologous *Carsonella* sRNAs have a potential molecular function, we conducted an evolutionary analysis to detect if signatures of selection were present in conserved sRNAs. Maximum-likelihood estimates of synonymous nucleotide divergence between all orthologous *Carsonella-BC* and *Carsonella-DC* proteins are saturated (185/188 have *dS* ≫ 3.0). Therefore, we compared the amino acid divergence of the corresponding protein coding region to that of the upstream and downstream flanking regions between *Carsonella-BC* and *Carsonella-DC* to examine the extent of asRNA conservation. In 8 out of the 11 cases, we found that the protein coding region corresponding to the asRNA was more conserved than the rest of the protein as a whole (one-tailed *t*-test, *P *<* *0.01; table 3).

To further investigate if conserved sRNAs are functional, we determined if the secondary structure of *Carsonella* sRNA orthologs was conserved and thermodynamically stable. Secondary structure and thermodynamic stability of sRNAs is important for their function with the stem and loop of a hairpin being one of the most common structures (Svoboda and Di Cara 2006; Weinberg et al. 2010; Małecka et al. 2015; Stav et al. 2019). Overall, the aligned regions of conserved asRNAs identified in both *Carsonella-BC* and *Carsonella-DC* were predicted to have multiple hairpins with multiple predicted compensatory changes that conserve the structure of the sRNA (table 3).

To determine whether these conserved asRNAs may be conserved with other sequenced *Carsonella* strains, we analyzed alignments of the homologous gene regions of *Carsonella-BC*, *Carsonella-DC*, and nine additional *Carsonella* genomes (see Materials and Methods). All of the genes encoding conserved asRNAs were present in the nine genomes, except for *Carsonella*-*CE* (NC_018414) and *Carsonella*-*CS* (NC_018415) that have lost the *carAB* operon (Sloan and Moran 2012). In each case, the overall predicted structure from the multigenome alignment had a lower thermodynamic ensemble prediction (kcal/mol) than the pairwise structures (table 3). However, in each case a similar number of compensatory changes were detected, and we could detect as much as 53% of the sRNA predicted interacting basepairs conserved between the pairwise sRNA structure and the multigenome sRNA structure (table 3). Together, these data indicate that some of the asRNAs are broadly conserved and possibly expressed among a wide diversity of *Carsonella* strains.

### *Carsonella-BC* sRNAs Are Differentially Expressed between *B. cockerelli* Life Stages

Though the endosymbionts exist in a relatively stable intracellular environment within their hosts, there are various developmental time points where symbionts encounter dynamic environmental changes, such as the period when they are vertically transmitted from adults to offspring. Previous research has shown that *Buchnera* differentially expresses its sRNAs and their protein targets between the embryonic and maternal bacteriocyte life stages in asexual parthenogenic aphids (Hansen and Degnan 2014; Thairu et al. 2018; Thairu and Hansen 2019a). In contrast to parthenogenic aphids, psyllids are sexual and during the adult stage of psyllid development, bacteriocytes undergo structural changes, and in females, migrate to the ovaries where *Carsonella* is transferred (Dan et al. 2017). This marks a shift in environment compared with all other nymphal life stages were *Carsonella* is only found within the bacteriocytes. Here, we compared sRNA expression between 5th nymphal instar psyllids which do not have fully developed sexual organs and the bacteriocytes of adult psyllids.

Small-RNA profiles were examined between two different life stages of *Carsonella-BC*, adults (BC-A1-3), and 5th instar nymphs (BC-N1-3) to determine if differential expression of *Carsonella* sRNAs occur between these two stages of development. Although RNAseq coverage was 66× lower for BC-N1-3 samples compared with the BC-A1-3 samples only differentially expressed sRNAs with significant normalized *q* values (false discovery rate [FDR] adjusted *P* values) of *q *≤* *0.05 were evaluated that also had an average of ≥9 reads across all three replicates (table 1). Expression analysis revealed that of the 36 asRNAs identified in *Carsonella-BC*, four were significantly upregulated in the adults, whereas none were significantly upregulated in 5th instar nymphs (supplementary table 3, Supplementary Material online). Two of the asRNAs upregulated in the adults were predicted to target CDSs in the three PANTHER GO pathways of arginine, leucine, and de novo pyrimidine ribonucleotide biosynthesis (table 4).

**Table 4.** GO PANTHER Pathways of the Predicted CDSs for sRNAs of *Carsonella-BC* That Are Differentially Expressed between the Adult (BC-A1-3) and Nymph (BC-N1-3) Samples.

	Predicted CDS of Expressed sRNA	
Pathway Associated with Predicted sRNA Target	Predicted CDS of Differentially Expressed sRNA *Carsonella-BC*	Life Stage That sRNA Is Upregulated
Arginine biosynthesis	*carA-carB*	Adult (BC-A1-3)
De novo pyrimidine ribonucleotides biosynthesis	*carA-carB*	Adult (BC-A1-3)
Leucine biosynthesis	*leuC*	Adult (BC-A1-3)

Note.—Underlined pathways are related to essential amino acid biosynthesis.

### Profftella Expresses Both Antisense and Intergenic sRNAs

*Profftella* belongs to a different class of bacteria than *Carsonella*, the Betaproteobacteria, and appears to have cospeciated with both *Carsonella* and psyllids within the psyllid genus of *Diaphorina* (Nakabachi, Malenovský, et al. 2020; Nakabachi, Piel, et al. 2020). sRNAs from *Profftella* were simultaneously isolated from *D. citri’s* bacteriome. Using Rockhopper, 181 asRNAs and four intergenic sRNAs were found to be expressed by *Profftella* (supplementary table 4, Supplementary Material online). No putative UTR sRNAs were detected. Notably, 16 of the asRNAs (*dipE_1-2*, *dipJ*, *dipO*, *dipP_1-2*, *dipQ*, *dipR_1-2*, and *dipT_1-7*) were predicted to target seven CDSs in the polyketide synthase biosynthetic gene clusters (supplementary tables 4 and 5, Supplementary Material online). The polyketide synthase biosynthetic genes are responsible for the production of the toxin, diaphorin (Nakabachi et al. 2013).

### AT Richness, GC Skew, and Conserved Motifs Are Present in the Promoter Regions Upstream of Expressed sRNAs

Currently, it is unclear how putative asRNAs are potentially regulated in host-restricted genomes of bacterial endosymbionts. To increase our understanding of potential mechanisms of regulation, we investigated if AT richness, GC skew, and conserved motifs were associated with predicted promoter regions of sRNAs. First, regions upstream of expressed asRNAs in *Carsonella*, *Profftella*, *Buchnera*, and *E. coli* were analyzed for a reduction in the percentage of GC (i.e., increased AT richness) compared with randomized sequences because a decrease in GC percentage can indicate a potential sRNA promoter site (Meysman et al. 2014). Within the 61-nt upstream region of asRNAs across all symbiont genomes surveyed and *E. coli*, there were regions that had a significant difference in GC percentage compared with randomized sequences (*P *<* *0.05, fig. 1 and supplementary table 6, Supplementary Material online). Specifically, all taxa except *Buchnera*-*UA* displayed a reduction in GC percentage between windows 33–48 which corresponds to 32–60 nt upstream of the sRNA putative transcriptional start sites. In addition, taxa that are more closely related to one another appear to share similar nt windows for reductions in GC percentage. For example, *Buchnera* lineages (*Buchnera 5A* and *AK*) that belong to the same aphid host genus and are more closely related to one another compared with other *Buchnera* taxa analyzed in this study both display significant reductions in the percentage of GC at three main upstream regions. The first reduction occurs between 1 and 16 nt, the second reduction occurs between 23 and 32 nt, and third reduction occurs between 45 and 53 nt upstream of their asRNAs. Significant increases in GC percentage were also observed. For example, three highly divergent taxa *Carsonella*-*BC*, *Buchnera*-*SG*, and *E. coli* increase in the percentage of GC between 17 and 21 nt upstream of their asRNAs.

**Fig. 2.**
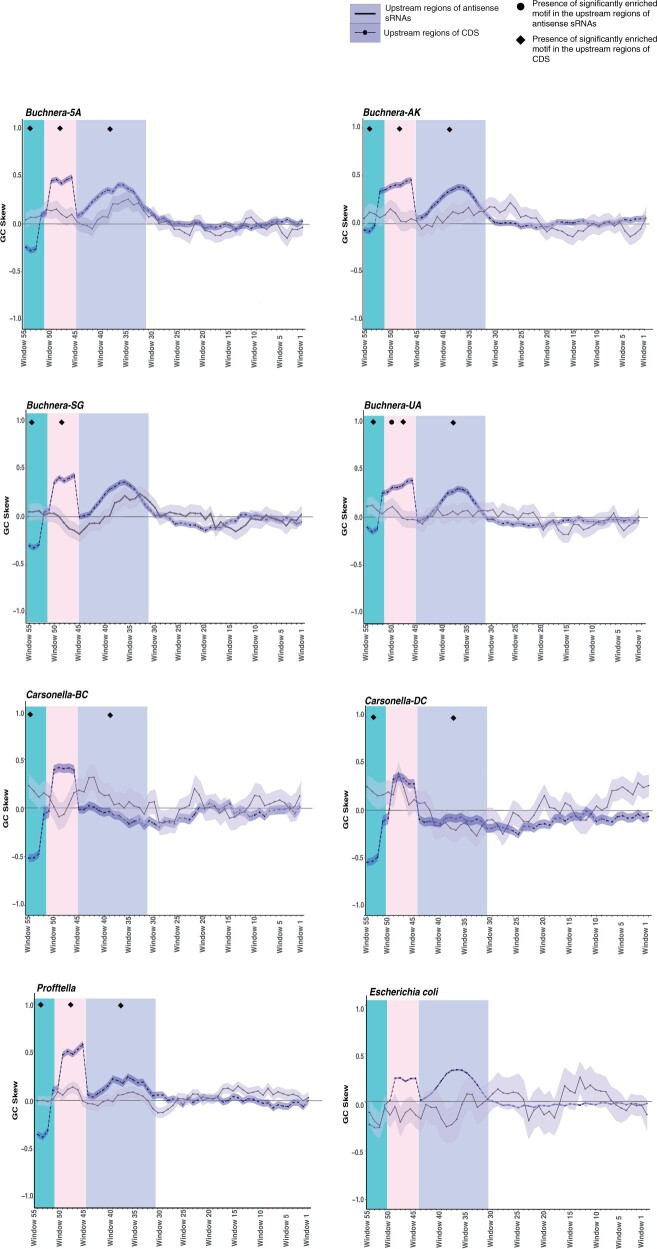
Sliding window analysis of the GC skew found in the 61 nt upstream region of expressed asRNAs and coding sequences in symbiont lineages and *Escherichia coli*. The shaded region around each line represents the standard error. Sample sizes: *Buchnera 5A*: CDS = 558, asRNAs = 90; *Buchnera-AK*: CDS = 569, asRNA = 70; *Buchnera-SG*: CDS = 545, asRNAs = 73; *Buchnera-UA* CDS = 539, asRNAs = 76; *Carsonella-BC*: CDS = 196, asRNAs = 36; *Carsonella-DC*: CDS = 207, asRNAs = 33; *Profftella:* CDS = 367, asRNAs = 181; *E. coli:* CDS = 4,140, asRNAs = 16.

Second, the patterns of GC skew were qualitatively compared across taxa because statistics need not be applied as the entire set of upstream regions that followed our selection criteria were included in the analysis. This inspection revealed three regions with conspicuous deviations in GC skew upstream of CDSs (fig. 2). In all eight genomes between windows 53 and 55 (green highlighted region corresponding to 53–61 nt upstream), a major decrease in GC skew was observed (fig. 2). Immediately adjacent to this, two regions of increased GC skew were identified. The first region between windows 46 and 54 (pink highlighted region corresponding to 46–54 nt upstream) was also identified in all eight genomes (fig. 2). However, the second region of increased GC skew windows 31 and 44 (purple highlighted region corresponding to 31–50 nt upstream) was detected in all taxa except for the two smallest genomes *Carsonella-DC* and *Carsonella-BC* (fig. 2). Similar changes in GC skew were not as widespread and pronounced in the regions upstream of the corresponding asRNAs, including previously characterized asRNAs from *E. coli* (fig. 2). Slight peaks in GC skew were detected in *Buchnera-5A*, *Buchnera-AK*, *Carsonella-DC*, and *Profftella* between windows 46 and 54, and only *Buchnera-5A*, *Buchnera-SG*, and *Profftella* showed isolated peaks between windows 31 and 44 (fig. 2).

Third, the Multiple Expectation maximizations for Motif Elicitation (MEME) tool, which detects conserved sequence patterns or motifs (Bailey et al. 2009), was used to determine if regions with deviations in GC content or GC skew correspond to conserved sequence motifs that may be acting as promoter sequences for asRNA or CDS expression. When the entirety of the asRNA upstream regions were analyzed for each genome, a single significantly enriched motif was detected in 8–62% of the upstream sequences of asRNAs from the *Carsonella-BC*, *Buchnera-5A*, *Buchnera-AK*, *Buchnera-UA*, and *Profftella* genomes (table 5 and supplementary table 7*A*, Supplementary Material online). Pooling the genomes together at the genus or symbiont taxa level also identified long conserved motifs in as many as 45% of the sequences from all of the genomes (table 5 and supplementary table 8, Supplementary Material online). Subsequent attempts to partition the alignments and only analyze the regions with changes in GC skew or significant differences in GC content identified motifs, however, none were significantly enriched (supplementary tables 7*B* and 7*C*, Supplementary Material online). In contrast, analysis of the upstream regions of CDSs with the observed changes in GC skew, did identify significantly enriched and conserved sequence motifs in two or more of these regions in all genomes (supplementary table 7*D* and fig. 2A, Supplementary Material online). These three regions of pronounced GC skew were then pooled among all of the symbionts, which revealed motif conservation in the upstream regions of CDS in the majority of the genomes (supplementary tables 7*E* and 8 and fig. 2*B*, Supplementary Material online). Conserved motifs were also found in the upstream regions of CDSs across all three regions of interest when *E. coli* was included (supplementary table *7F* and fig. 2*B*, Supplementary Material online).

**Table 5.** Significantly Enriched Motifs Identified in the Upstream Regions of Expressed sRNAs Identified in *Carsonella*, *Profftella*, and *Buchnera* Genomes. 2 Carsonella-BC

Genome(s)	No. of sRNA Upstream Regions Searched	Motif^a^	*E*-value	% of Sequences w/Motif
Species-level motifs				
*Carsonella*-*BC*	34	GATWWWKTAAHAAWWKBAGSW	3.7E-05	62%
*Profftella*	159	YCASSWWBWHSWGMAATTGCWSMAGC	2.0E-05	8%
*Buchnera*-*5A*	89	ADMWDCAKYWWTWKYWDYTTTTTYT	8.3E-06	37%
*Buchnera*-*AK*	67	TATYTTCARWATTWBTWWYTTTTTTTTTD	2.4E-03	28%
*Buchnera*-*UA*	67	GCAWYARNTHCTGCT	1.1E-05	30%
				
Genus-level motifs				
*Carsonella*-*BC*,				
*Carsonella*-*DC*	65	TGVTRATTYARKAAWAVMWGCWTKAKCWD	3.6E-06	45%
*Buchnera*-*5A*	294	WAAWGCWAHTRHTTYTTTTTY	2.1E-37	24%
*Buchnera*-*AK*,				
*Buchnera*-*SG*,		CTGMTTTTAHTMTTSATVSWMMWATWYDRTWWGRTNYTGC	5.7E-46	5%
*Buchnera*-*UA*		AAAAAAAWMWACAAAWAWTRRADATAWWT	4.2E-24	15%
				
Symbiont-level motifs				
*Carsonella*-*BC*,				
*Carsonella*-*DC*,	518	HAGCWDYWGVTRYAKCWSCW	9.0E-33	9%
*Buchnera*-*5A*,				
*Buchnera*-*AK*,				
*Buchnera*-*SG*,		RCTGMTWTWAHTATTGMTVBWVVWATWMDRTWTGATGTTSC	3.9E-49	8%
*Buchnera*-*UA*				
*Profftella*		YTAAAAAAAWWAAARAAAAAR	2.2E-17	18%

a N = A/C/G/T, V = A/C/G, H = A/C/G, D = A/G/T, B = C/G/T, M = A/C, R = A/G, W = A/T, W = A/T, S = C/G, Y = C/T, K = G/T.

## Discussion

Our results provide further evidence that host-restricted bacterial lineages maintain conserved asRNA orthologs for millions of years, as bacterial taxa cospeciate with their host insects. Moreover, these conserved sRNA orthologs display signatures of selection, conserved secondary structure, and differential expression between host life stages further indicating that these conserved sRNAs are functional. In addition, we identified conserved patterns of AT and GC richness, GC skew, and sequence motifs upstream of expressed asRNA in the host-restricted genomes of *Carsonella*, *Buchnera*, and *Profftella*. These conserved nucleotide composition patterns and motifs may be involved in sRNA regulation by modifying DNA secondary structure and/or providing binding sites for RNA polymerase or putative regulatory protein(s).

A consequence of genome shrinkage experienced by intracellular bacteria is a marked increase in AT content particularly within intergenic spacers and synonymous sites within CDSs (McCutcheon and Moran 2011). *Carsonella* and *Profftella* are no exception to this rule having ∼84% and ∼76% AT, respectively (Nakabachi et al. 2013; Riley et al. 2017). This genome-wide AT bias often confounds promoter scans using motifs based on characterized binding sites from *E. coli*, given the AT richness of the *E. coli* consensus motifs (Huerta et al. 2006). Thus, it has been suggested that the high frequency of asRNA expression that is observed in endosymbiont genomes is the result of erroneous transcriptional activity due to the high presence of AT-rich promotor-like motifs such as the Pribnow motif (TANAAT) which generally occurs ∼10 bp upstream of the initiation of transcription (Lloréns-Rico et al. 2016). The results of the sliding window analysis performed here demonstrated that the majority of the taxa in this study display significantly lower percent GC between 32 and 60 nt upstream of sRNA expression indicating that an AT-rich promoter at ∼10 bp upstream does not occur for all sRNAs in the majority of taxa. The differences in GC skew in the upstream regions of CDSs and asRNAs further support the hypothesis that if cryptic regulatory regions exist in these upstream regions of asRNAs, they are unique from the regulatory regions found in CDSs (fig. 2). Further such sequence variation is key to determining bacterial promoter strength and is critical for fine tuning gene regulation (Bervoets and Charlier 2019). As such, asRNAs may be expressed at levels below that or under different conditions relative to their cognate CDSs.

*Buchnera* is one of the few nutritional endosymbionts like *Carsonella* that has had its sRNAs interrogated (Thairu and Hansen 2019b). Genomes of *Buchnera* from four divergent aphid species were characterized and a total of ∼236 asRNAs from each species were identified, 115 of which were conserved in two or more taxa (Hansen and Degnan 2014). In the current study, we have identified an average of 34 asRNAs per *Carsonella* taxa. This is a similar asRNA density between these divergent symbionts given the difference in genome size and gene number; the genome sizes and gene number of the *Carsonella* taxa used in this study are ∼1/4 the size of the *Buchnera* taxa previously analyzed (table 1) (Hansen and Degnan 2014). Although comparative genomic studies indicate that UTR-encoded sRNAs are lost as genomes shrink (Matelska et al. 2016), over 500 conserved UTR or intergenic associated sRNAs were still detected in *Buchnera* genomes (Hansen and Degnan 2014)*.* This is not the case in *Carsonella*, as genomes did not have any identifiable intergenic sRNAs because there are in fact very few intergenic regions (Sloan and Moran 2012). In addition to *Carsonella*, we also identified sRNAs for the first time in *Profftella* the defensive co-symbiont of *D. citri* that is similar in genome size to *Buchnera.* Both antisense and intergenic sRNAs were identified from *Profftella*, seven of which are predicted to target CDSs in the biosynthetic gene clusters responsible for the production of the protective toxin, diaphorin, which has been shown to be toxic to potential psyllid natural enemies (Yamada et al. 2019).

Although *Carsonella-BC* and *Carsonella-DC* represent two symbiont lineages from divergent hosts, 11 sRNAs that are expressed antisense to the CDSs *aroC*, *atpA*, *atpD*, *carA*, *carB*, *clpX*, *gidA*, *grepE*, *leuC*, and *prfA* are conserved between them*.* Five of these CDSs *clpX*, *carB*, *gidA*, *grpE*, and *prfA* are also regulated by conserved asRNAs in *Buchnera* (Hansen and Degnan 2014). Though both *Carsonella* and *Buchnera* are Gammaproteobacteria and Hemipteran endosymbionts, evidence suggests that they are not closely related (Williams et al. 2010; Lang et al. 2013; Mondal et al. 2020). Also, both symbionts have coevolved with their hosts that diverged ∼300–350 Ma further supporting the hypothesis that these symbionts have greatly diverged from each other (Thao et al. 2001; Peccoud et al. 2009; Nováková et al. 2013; Hall et al. 2016; Johnson et al. 2018). The conserved *Buchnera* asRNA for *carB* was shown to activate/or stabilize its predicted gene target when heterologously expressed in *E. coli* (Thairu et al. 2018). These results were also corroborated in vivo as the *Buchnera* asRNA *carB i*s upregulated in aphid ovarioles, the same life stage that the protein, CarB, is upregulated in comparison to maternal bacteriocytes (Hansen and Degnan 2014; [Bibr msab202-B69][Bibr msab202-B70]). If all of these sRNAs are functional, this finding suggests that these distinct symbiont species that have coevolved in two different insect superfamilies may have convergently evolved regulatory sRNAs to target the same CDSs from different locations within the CDS. These conserved sRNAs from *Carsonella* and *Buchnera* represent key targets for future functional studies.

In both *Buchnera* and *Carsonella* taxa, not all sRNAs detected were conserved and possible sequence motifs were not universally shared by all analyzed genomes within a particular lineage. This observation could be partially an artifact of sequence coverage; however, it also is suggestive of lineage-specific gains or losses of sRNAs. Furthermore, this phenomenon is widely observed among characterized microbes given the lability, and economy of RNAs that allows microbes to rapidly respond to changes in the environment (Beisel and Storz 2010). Thus, the variability in sRNAs detected could be an indicator of on-going evolutionary changes in both *Buchnera* and *Carsonella* even though their genomes are largely stable and syntenic. Nevertheless, we note that our current estimates of *Carsonella* sRNAs likely represent lower bounds on both the overall number of sRNAs as well as those which are differentially regulated. Additional trials testing a variety of environmental conditions and life stages of psyllids will most likely result in the identification of more putative sRNAs within *Carsonella*.

In this study we also demonstrated that sRNAs are differentially expressed between the late nymphal and adult life stages of *Carsonella-BC*, including sRNAs that are predicted to target genes within the essential amino acid biosynthesis pathways for leucine and arginine. Life stage differences in symbiont sRNA expression has also been observed in *Buchnera* taxa and has been associated with differential expression of their protein targets (Hansen and Degnan 2014; Thairu et al. 2018; Thairu and Hansen 2019a). These results indicate that symbionts with reduced genomes may rely on sRNA regulation in response to their host’s dynamic nutritional demands for essential amino acids throughout insect development (Rabatel et al. 2013; Pers and Hansen 2021). This is an important finding because many of these symbionts have lost key regulatory elements and genes, including canonical protein encoded regulatory mechanisms (Thairu and Hansen 2019b). Nevertheless, there is increasing evidence that host-restricted symbionts, and organelles that share similar genomic characteristics, use regulatory sRNAs (Thairu and Hansen 2019b).

With the increase of “omics”-based experiments, there is emerging evidence that sRNAs are expressed within highly reduced, host-restricted bacterial genomes, and these sRNAs have functional roles in gene regulation (Dietrich et al. 2015; Thairu and Hansen 2019b). Though sRNAs are known to be important in bacterial gene regulation, we speculate that the evolution of regulatory sRNAs in small bacterial genomes generally occurs as a compensatory or perhaps adaptive mechanism to regulate key symbiotic and core housekeeping genes that have lost their regulators through genome reduction processes. Given the rapid nature of sRNA evolution, we predict that this type of gene regulation can keep up with higher rates of mutation that occur in host-restricted bacterial symbionts that are obligate. Overall, we hypothesize, that when bacterial genomes lose protein regulators, host-restricted genomes revert to an “RNA world” of gene regulation. Based on evidence from other systems where bacterial symbionts and organelles have reduced genomes (Thairu and Hansen 2019b), we predict that some of the sRNAs identified here will be borne out to be functional.

## Materials and Methods

### Small RNA Sample Preparation and Sequencing

*Bactericera cockerelli* and *D. citri* psyllids were reared at ∼27 °C under a 16-h light/8-h dark regime on 6–12-week-old tomato (*Solanum lycopersicum*) and ∼1-year-old curry leaf (*Murraya koenigii*) plants, respectively. For *B. cockerelli*, three samples were collected (BC-A), (BC-N), and (BC-All). For the first and second samples (BC-A and BC-N), three biological replicates of ∼60 psyllids (approx. 30 males and 30 females per sample) were collected. For the third, samples from both psyllid species (BC-All and DC-All) consisted of a mixed population of ∼20 psyllids per life stage and were collected and combined into a single sample per species. All tissues were immediately placed in RNAprotect Bacteria Reagent (Qiagen, Germantown, MD) and stored at −80 °C.

For each sample, RNA was extracted using the Quick-RNA Microprep kit (Zymo, Irvine, CA). Library preparation and sequencing was then performed on the sRNA-enriched fraction (≤250 nt) using the Illumina mRNA strand-specific sequencing protocol by the University of California, San Diego, Institute for Genomic Medicine Genomics Center (UCSD IGM Genomics Center). Each library was then sequenced as 75 nt single-end reads on the Illumina Hi-seq 4000 (San Diego, CA, USA) at the UCSD IGM Genomics Center. All sequence data from this study were submitted under NCBI bioproject ID: PRJNA562893.

### Identification and Categorization of *Carsonella* and *Profftella* sRNAs

For all samples, reads were quality screened and adapters were removed using Trimmomatic v.0.33 (Bolger et al. 2014) and Cutadapt v2.1 (Martin 2011). For *B. cockerelli* samples, reads mapping to *Carsonella-BC* were aligned using Bowtie2 v.2.2 (Langmead et al. 2009). Bowtie2 v.2.2 was also used to map reads from the *D. citri* sample to either *Carsonella-DC* or *Profftella.* Rockhopper v.2.0.3 (McClure et al. 2013) was then used to identify putative *Carsonella* and *Profftella* sRNAs. To maximize detection of conserved sRNAs between the *Carsonella* lineages, the parameters of Rockhopper for the “minimum expression of untranslated regions (UTR) and non-coding RNAs (ncRNA)” was set at 0.3. All other parameters were left at the default settings for strand-specific reads. Using the default parameters for strand-specific reads in Rockhopper putative sRNAs were identified in *Profftella* as well. All symbiont sRNAs were then binned into three different categories: sRNAs expressed antisense to the gene (asRNAs), sRNAs expressed within the untranslated regions of genes (UTR sRNAs), and sRNAs identified within the intergenic spacer regions (intergenic sRNAs). To determine the effect of read sampling on sRNA detection, the *Carsonella-BC* data set was randomly resampled in triplicate, analyzing 90%, 75%, 50%, 25%, 10%, 5%, 2.5% and 1% of the reads in Rockhopper using the same parameters as described above (supplementary fig. 1, Supplementary Material online).

Rockhopper was also used to determine if *Carsonella-BC* sRNAs were differentially expressed between the two life stages, adult (samples: BC-A1-3) and 5th instar nymphs (BC-N1-3). Rockhopper normalizes reads among samples using the upper-quantile normalization method (McClure et al. 2013).

### Identification and Analysis of Conserved *Carsonella* sRNAs

To determine whether any of the asRNAs detected were expressed from orthologous genomic locations, the genomes of *Carsonella-BC* and *Carsonella-DC* were aligned with progressiveMauve (Darling et al. 2010). Using the alignment information, a custom PERL script was used to identify overlapping or adjacent Rockhopper predicted asRNAs. Orthologous locations were confirmed by aligning the DNA sequences of open reading frames containing the asRNA, based on their amino acid sequences in Muscle (Edgar 2004) and manually adding the asRNAs to the alignment in Mesquite (Maddison and Maddison 2019). Conserved asRNAs were defined as having Rockhopper predicted coordinates within 15 nt of one another and continuous RNAseq read coverage as visualized in Artemis v.16 (Rutherford et al. 2000). Secondary structure predictions were generated for the *Carsonella-BC* and *Carsonella-DC* regions encompassing both sRNAs and extending 15 nt upstream and 15 nt downstream using RNAalifold (Bernhart et al. 2008) following the methods of Hansen and Degnan (2014). Conserved asRNA regions were further analyzed by including orthologous regions from nine other *Carsonella* genomes, and similarly folded with RNAalifold. This included *Carsonella-JRPAMB4* (NZ_CP041245), *Carsonella-YCCR* (NZ_CP012411), *Carsonella-CE* (NC_018414), *Carsonella-CS* (NC_018415), *Carsonella-PC* (NC_018418), *Carsonella-PV* (NC_008512), *Carsonella-BT* (NZ_CP024798), *Carsonella-HC* (CP003543), and *Carsonella-HT* (NC_018417).

Furthermore, pairwise maximum-likelihood amino acid sequence divergence of the coding regions encompassed by the asRNAs were compared with that of the flanking regions for each of the conserved sRNAs between *Carsonella-BC* and *Carsonella-DC* using AAML in PAML v3.9 (Yang 2007). Given the marked size difference in the sRNAs, their flanking regions, and the effect sequence length can have on divergence estimates, a sliding window approach was used for each gene to generate a distribution of amino acid sequence divergence estimates. Each divergence estimate for the sRNA region was then compared with this distribution with a one-tailed *t*-test in JMP Pro v13 to identify patterns of conservation. To determine whether conserved sRNAs represented a significant proportion of the sRNAs identified in each genome, a one-sided *z*-proportion test was used, in which the proportion of conserved sRNAs was compared with the proportion of sRNAs found per CDS.

### Identification of Putative sRNA Promoter Regions

To further understand the potential mechanisms of asRNA expression and regulation within intracellular symbiont genomes, we searched the upstream regions of expressed sRNA for AT richness, GC skew, and enriched motifs, which may act as potential promotor sequences. We focused on asRNAs for the following upstream analyses because asRNAs were the most abundant sRNAs identified across all taxa examined in this study. For these analyses, asRNAs identified in both *Carsonella* lineages, *Profftella*, as well as asRNAs identified by Hansen and Degnan (2014) in *Buchnera* from the aphid species *Acyrthosiphon pisum* (*Buchnera*-*5A*), *Acyrthosiphon kondoi* (*Buchnera*-*AK*), *Uroleucon ambrosiae* (*Buchnera-UA*) and *Schizaphis graminum* (*Buchnera*-*SG*), and the asRNAs identified in *Escherichia coli* were used (Shinhara et al. 2011; Rau et al. 2015; Keseler et al. 2017). Using a sliding window approach (each window was 7 nt with a 1-nt step size) within a 61-nt region upstream of each sRNA, the nucleotide composition, defined as percent GC and GC skew ([C − G]/[C + G]) were determined.

To determine whether the percentage of GC was significantly different for each window, the upstream region of each asRNA was re-shuffled 100 times to create a null distribution. A *t*-test was then performed by comparing the percent of GC in the given sequence to that of the mean of the reshuffled upstream region for each window. However, if the upstream regions overlapped an adjacent coding sequence (CDS), then that asRNA was dropped from the GC percent analysis. To determine whether a pattern of GC skew occurs in the upstream region of each asRNA, GC skew was compared in the upstream region of all asRNAs identified (61 nt) within a symbiont’s or *E. coli’s* genome to upstream region of all CDSs (61 nt) within each respective genome. When comparing the GC-skew patterns of the upstream regions of asRNAs and the CDSs, all known asRNAs and CDSs were included if the respective 61 nt upstream regions did not overlap an adjacent CDS. Because the entire population of upstream regions that fit the criteria was included in the analysis, not a subset of a population, no further statistical tests were needed.

To find potential sequence motifs, upstream of each sRNA MEME (Bailey et al. 2009) was used for each window 61 nt upstream of an asRNA, with default settings to identify potential motifs >4 nt long. The potential presence of conserved motifs both within the same genus and across different taxa were investigated.

## Supplementary Material

Supplementary data are available at *Molecular Biology and Evolution* online.

## Supplementary Material

msab202_Supplementary_Data
