# Supplementary material for: Natural Selection Shapes Maintenance of Orthologous sRNAs in Divergent Host-Restricted Bacterial Genomes
Source: Mol Biol Evol. 2021 Jul 2;38(11):4778–91. doi: 10.1093/molbev/msab202 (PMC8557413; doi:10.1093/molbev/msab202)
Supplement: msab202_Supplementary_Data [file msab202_supplementary_data.zip › Supplementary Figures.docx]

**Supplementary Figure 1:** Rarefaction analysis of the 6.1 million reads recovered from the 7 *Carsonella-BC* samples. Each sample was randomly sub-sampled three times and 90%, 75%, 50%, 25%, 10%, 5%, 2.5% and 1% of the reads were analyzed with Rockhopper. The plot shows the number of the 36 *Carsonella-BC* antisense sRNAs detected using the subsampled datasets. ≥79% of the *Carsonella-BC* antisense sRNAs are successfully identified using as few as 2.5% of the reads (153,000). For comparison the total number of reads recovered for *Carsonella-DC* (red star) and *Profetella* (blue star) are shown on the secondary X-axis.


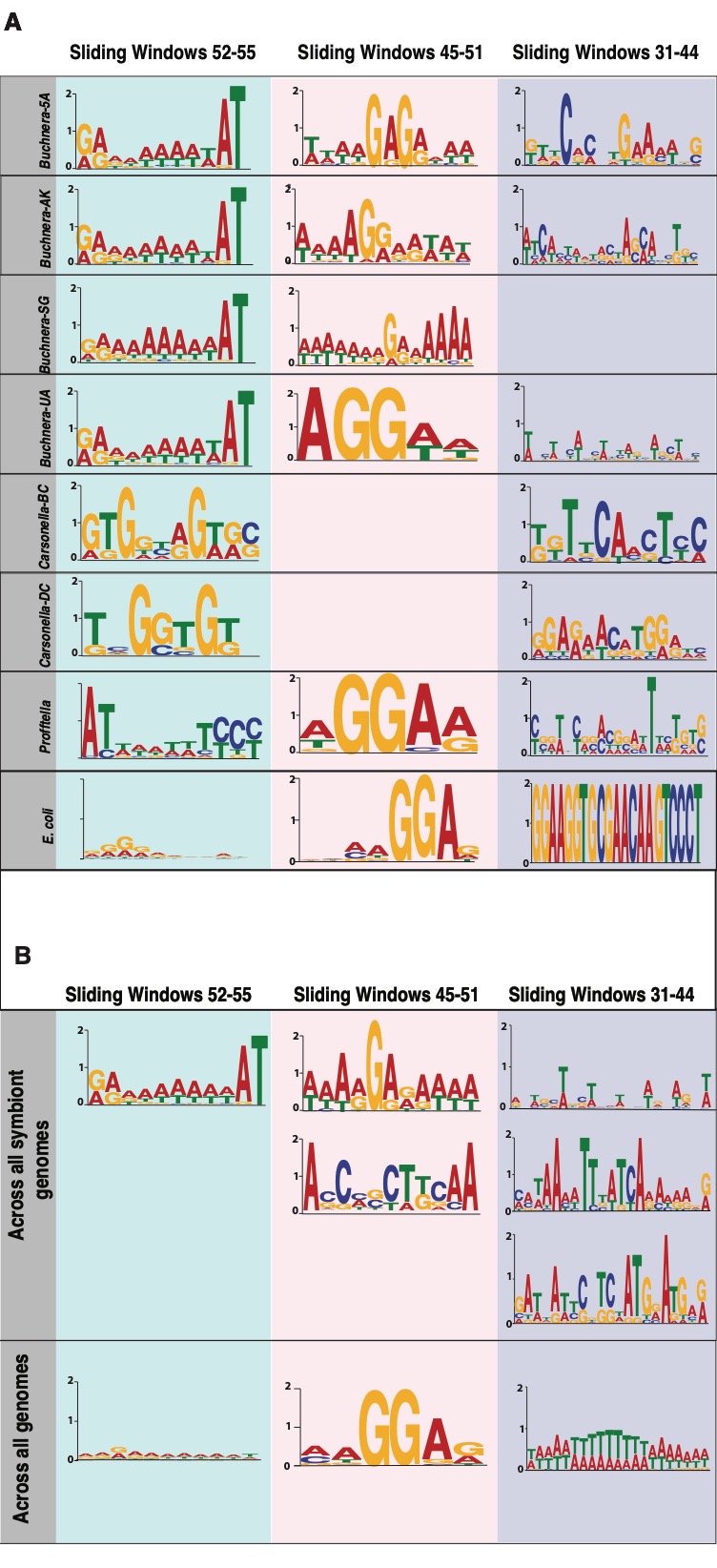


**Supplementary Figure 2**: Logograms of enriched motifs identified (E <0.05) in the upstream region of expressed antisense sRNAs across symbiont and *E.coli* genomes**. 1A**: Enriched motifs found in the 61nt upstream regions of expressed antisense sRNAs. **1B**: Enriched motifs found within the 61nt upstream region of expressed antisense sRNAs found in either both *Carsonella* lineages or the five *Buchnera* lineages analyzed. **1C:** Enriched motifs found within the 61nt upstream region of expressed antisense sRNAs found across *E.coli* and all symbiont genomes surveyed: both *Carsonella* lineages, the five *Buchnera* lineages*,* and *Profftella*.
